# Cellular senescence is a double‐edged sword in regulating aged immune responses to influenza

**DOI:** 10.1111/acel.14162

**Published:** 2024-04-30

**Authors:** Blake L. Torrance, Andreia N. Cadar, Hunter A. Panier, Dominique E. Martin, Erica C. Lorenzo, Evan R. Jellison, Jenna M. Bartley, Laura Haynes

**Affiliations:** ^1^ UConn Center on Aging University of Connecticut School of Medicine Farmington Connecticut USA; ^2^ Department of Immunology University of Connecticut School of Medicine Farmington Connecticut USA; ^3^ Department of Medicine University of Connecticut School of Medicine Farmington Connecticut USA

**Keywords:** aging, influenza, memory, p16, senescence, T cells

## Abstract

Clearance of senescent cells has demonstrated therapeutic potential in the context of chronic age‐related diseases. Little is known, however, how clearing senescent cells affects the ability to respond to an acute infection and form quality immunological memory. We aimed to probe the effects of clearing senescent cells in aged mice on the immune response to influenza (flu) infection. We utilized a p16 trimodality reporter mouse model (p16‐3MR) to allow for identification and selective clearance of p16‐expressing cells upon administration of ganciclovir (GCV). While p16‐expressing cells may exacerbate dysfunctional responses to a primary infection, our data suggest they may play a role in fostering memory cell generation. We demonstrate that although clearance of p16‐expressing cells enhanced viral clearance, this also severely limited antibody production in the lungs of flu‐infected aged mice. 30 days later, there were fewer flu‐specific CD8 memory T cells and lower levels of flu‐specific antibodies in the lungs of GCV‐treated mice. Furthermore, GCV‐treated mice were unable to mount an optimal memory response and demonstrated increased viral load following heterosubtypic challenge. These results suggest that targeting senescent cells may potentiate primary responses while limiting the ability to form durable and protective immune memory with age.

## Graphical abstract

Deletion of p16‐expressing cells in a mouse model of flu infection improved viral clearance during a primary infection. However, it also decreased the generation of CD8 T‐cell memory and the levels of cross protective antibodies. This led to reduced protection following a subsequent infection. Targeting cellular senescence may potentiate primary immune responses but it comes at a cost to the generation of durable and protective immune memory.

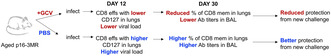

**Abbreviations**
BAL: bronchoalveolar lavage
DPI: days post infection
Flu: Influenza
FMO: fluorescence minus one
GCV: ganciclovir
HA: Hemagglutinin
MFI: Mean Fluorescence Intensity
MPEC: Memory Precursor Effector Cell
NA: Neuraminidase
NP: nucleoprotein
p16: p16INK4A
p16‐3MR: p16 trimodality reporter
PA: acid polymerase
PR8: H1N1 Influenza virus A/Puerto Rico/8/34
SASP: senescence associated secretory phenotype
SA‐β‐gal: Senescence Associated β‐galactosidase
SLEC: Short Lived Effector Cell
x31: H3N2 Influenza virus A/HKx31

## 1 INTRODUCTION

It is well appreciated that aging results in various changes in the immune system that leave older adults at higher risk for severe infection. This is demonstrated by the fact that older adults bear the greatest burden of adverse outcomes following both COVID‐19 and influenza (flu) infection (CDC, 2022, 2023). The effects of age on the immune system are multifaceted and affect both the innate (Shaw et al., 2010) and adaptive compartments (Frasca & Blomberg, 2020; Mittelbrunn & Kroemer, 2021). Both CD4 and CD8 T‐cell responses are also diminished with age. Following flu infection, aged mice, similar to older adults, have delayed viral clearance, reduced antibody responses, and a slower resolution of inflammation following pathogen clearance (Keilich et al., 2019). The mechanisms of T‐cell aging include both cell intrinsic and cell extrinsic factors (Mittelbrunn & Kroemer, 2021). Our group and others have described that the aged microenvironment is one of the key drivers of dysfunction in CD4 and CD8 T‐cell responses. Adoptive transfer of aged T cells into young hosts can rescue some of the age‐related changes such as reduced proliferative capacity, reduced cytotoxicity, and dysfunctional CD4 subset differentiation (Jiang et al., 2009; Lefebvre et al., 2012). Furthermore, with age, the capacity of T cells to effectively differentiate into protective memory cells is also compromised (Haynes et al., 2003). Since CD8 T cells are the key mediators of viral clearance, it is of utmost importance to understand how the aged microenvironment shapes their function, including the generation of protective T‐cell memory.

The aged microenvironment is characterized by a systemic increase in basal, sterile, and chronic inflammation, a state termed inflammaging (Franceschi et al., 2000). More recently, a role for senescent cells has emerged as a key factor in systemic increases in inflammation with age. Cellular senescence is a mostly irreversible state of cell cycle arrest that plays a key role during embryonic development and as a tumor suppression mechanism (Campisi & d'Adda di Fagagna, 2007). Senescent cells are also required for optimal wound healing (Demaria et al., 2014). After performing these physiological functions, senescent cells are typically cleared by the immune system. With age, however, these senescent cells accumulate, become resistant to apoptosis, and evade immune clearance. As these cells accumulate, they take on a heterogenous proinflammatory secretory program termed the senescence‐associated secretory phenotype (SASP) (Coppé et al., 2008). SASP also has the ability to induce senescence in nearby cells highlighting their pervasive nature leading to serious detrimental health outcomes (Acosta et al., 2013). Senescent cells have been linked to a wide variety of chronic diseases of aging in pre‐clinical mouse models. Osteoporosis, non‐alcoholic fatty liver disease, type 2 diabetes, and cardiovascular diseases have all been shown to improve following administration of senolytics, drugs that target senescent cells (Farr et al., 2017; Ogrodnik et al., 2017; Roos et al., 2016; Xu et al., 2015). The effects of senescent cells on homeostatic immune function in the absence of infection have revealed a complex relationship. As senescent cells emerge, the proinflammatory SASP can serve as a homing signal to immune cells to clear them (Sagiv & Krizhanovsky, 2013). This can compensate for the decreased efficacy of homeostatic immune surveillance, but there comes a point where the rate of senescent cell accumulation goes beyond the rate of clearance (Ovadya et al., 2018). This results in an accumulation of senescent cells that can contribute to the various pathologies that they are associated with. However, very few studies have focused on the role of senescent cells in exacerbating age‐related changes in the immune system during infection. Work by our group demonstrated that senolytic treatment is able to improve CD4 T‐cell subset balance following flu infection in aged mice (Lorenzo et al., 2022), while others have shown that senolytics improve primary responses to a mouse coronavirus (Camell et al., 2021).

The mechanistic link between senescent cells and T‐cell function remains unclear. Antiviral T‐cell responses require tight regulation to achieve robust proliferation upon antigen encounter, engagement of strong effector phenotypes to mediate viral clearance, development of memory precursors that differentiate into protective memory cells, and regulatory signals to mediate healing and return to homeostasis following viral clearance. Senescent cells and SASP may play pleiotropic roles, restraining aspects like initial effector function while potentiating others such as memory cell precursor differentiation. We aimed to investigate this by utilizing a powerful transgenic mouse model, the p16 Trimodality Reporter (p16‐3MR), which allows for identification of cells expressing p16^INK4a^ (p16), a key biomarker of senescent cells (Krishnamurthy et al., 2004), as well as selective clearance of these cells to understand how cellular senescence shapes immune responses with age.

## 2 METHODS

### 2.1 Mice

All experiments utilized aged (18–20mo) male and female p16‐3MR mice on a C57BL/6 genetic background (Demaria et al., 2014) which were bred and housed at UConn Health in specific pathogen free (SPF) conditions. Between 4 and 8 aged mice per treatment group were used. All mice underwent gross examination at the time of sacrifice, and animals with obvious pathology (e.g., tumors) were excluded from the study. Original breeding pairs were generously provided by Dr. Judith Campisi. All mice were housed in a climate‐controlled environment with a 12 hr light/12 hr dark cycle and fed standard chow [18 kcal% fat, 58 kcal% carbohydrate and 24 kcal% protein (Teklad global 18% protein rodent diet, ENVIGO, Indianapolis, IN)] and water ad libitum. All mice were cared for in accordance with the recommendations in the Guide for the Care and Use of Laboratory Animals of the National Institutes of Health. All procedures were approved by the UConn Health IACUC.

### 2.2 Ganciclovir (GCV) treatment

Mice were given 25 mg/kg/day ganciclovir (GCV, Acros Organics) dissolved in PBS via intraperitoneal (i.p.) injection. Control mice were injected with equal volume PBS. Mice were treated for five consecutive days as described (Demaria et al., 2014). Prior to infection, a 5 day wait period was observed to ensure full clearance of GCV. Pharmacokinetic studies have demonstrated that, upon i.p. administration, systemic GCV concentrations peak 1 hour after injection and are undetectable by 2 h (Boujemla et al., 2016).

### 2.3 Viral infection

Mice were placed under anesthesia with isoflurane and intranasally delivered sublethal doses of either H1N1 influenza virus A/Puerto Rico/8/34 (PR8) or H3N2 influenza virus A/HKx31 (x31). The primary infection dose for PR8 was 500 EID_50_ in 50 uL PBS. Rechallenge experiments utilized 700 EID_50_ in 70 uL of PBS to better probe the protection with a higher dose. For x31 infection, 3000 EID_50_ in 50 uL PBS was used. Mice were monitored daily to assess weight loss as an indication of infection progress. Moribund mice and mice that had lost more than 30% of their original body weight were euthanized.

### 2.4 Viral quantification

Following sacrifice, lungs were immediately flash frozen in liquid nitrogen. Lung tissue was homogenized using a handheld homogenizer (Pro Scientific) and RNA was isolated via standard trizol/chloroform (Invitrogen Life Technologies and Sigma Aldrich, respectively) extraction per the manufacturer's protocol. cDNA was synthesized using iScript cDNA synthesis kit (Bio‐Rad) using the manufacturers protocol. Viral load was determined by RT‐qPCR for PR8 acid polymerase (PA) gene compared to a standard curve of known PA copy numbers as we have previously published (Keilich et al., 2020). This method has been shown to directly correlate with other viral quantification methods (Jelley‐Gibbs et al., 2007). The following primer and probe were used: forward primer, 5′‐CGGTCCAAATTCCTGCTGA‐3′; reverse primer, 5′‐.

CATTGGGTTCCTTCCATCCA‐3′; probe, 5′‐6‐FAM‐

CCAAGTCATGAAGGAGAGGGAATACCGCT‐3′ (Integrated DNA Technologies).

### 2.5 Tissue processing and flow cytometry

Following sacrifice, lungs were mechanically and enzymatically digested (100 U/mL collagenase (Gibco)) in RPMI media containing 5% fetal bovine serum. Red blood cells were lysed using ACK lysis buffer (Gibco). Spleens were mechanically digested through 70 um filters, and red blood cells were lysed using ACK buffer. Lymph nodes were mechanically digested through 70 um filters and did not undergo red blood cell lysis. All cell count calculations were based on the original count of total live cells in each single cell suspension.

For flow cytometry, cells were incubated with Fc block (anti‐CD16/32, Thermo Fisher) followed by staining with a NP_311‐325_ IA^b^ MHC Class II tetramer or NP_366‐374_ H‐2D^b^ MHC Class I tetramer (generated by the NIH Tetramer Core Facility). Cells were subsequently stained with surface antibodies and then either fixed using 1% paraformaldehyde or permeabilized using a FoxP3/Transcription factor fixation/permeabilization kit (Thermo Fisher). Samples undergoing permeabilization were then stained with intracellular antibodies. Extended antibody information can be found in Table S1. The PE channel was always kept clear for analysis of RFP expression by p16^+^ cells. Becton Dickinson (BD) LSR II or Bio‐Rad ZE5 cytometers were used, and analysis was performed using FlowJo (BD). Fluorescence minus one (FMO) controls were used for all markers.

### 2.6 Antibody quantification

To obtain bronchoalveolar lavage (BAL), at time of sacrifice, lungs were flushed with 1 mL of PBS and supernatant was collected following centrifugation to exclude cells and debris. Serum was obtained from blood collected via cardiac puncture immediately postmortem. Serum samples were serially diluted 10‐fold. BAL samples were initially diluted 1:500 and then serially 1.75 fold. Diluted samples were transferred to microplates coated with either whole viral particle or flu nucleoprotein (NP). Depending on the assay, either a horseradish peroxidase conjugated to an anti‐IgG or anti‐IgA antibody (Southern Biotech) was used. Titer was determined at highest dilution which had a measured absorbance at 490 nm over mean plus standard deviation of blanks. Two technical replicates were performed, and the average across both was taken to determine the sample titer.

### 2.7 Statistics

All data are presented as mean +/− standard error of the mean (SEM). Outliers were detected using Grubb's test. This resulted in the exclusion of one high outlier in the PBS group in Figure 4b. Differences between groups were determined via Student's t‐test or Mann–Whitney *U*‐test when data were not normally distributed as indicated by the Shapiro–Wilk test. Analyses were performed using Prism 8 software (GraphPad). *p* values <0.05 were considered significant (*p < 0.05, **p < 0.01).

## 3 RESULTS

3.1

**FIGURE 1:**
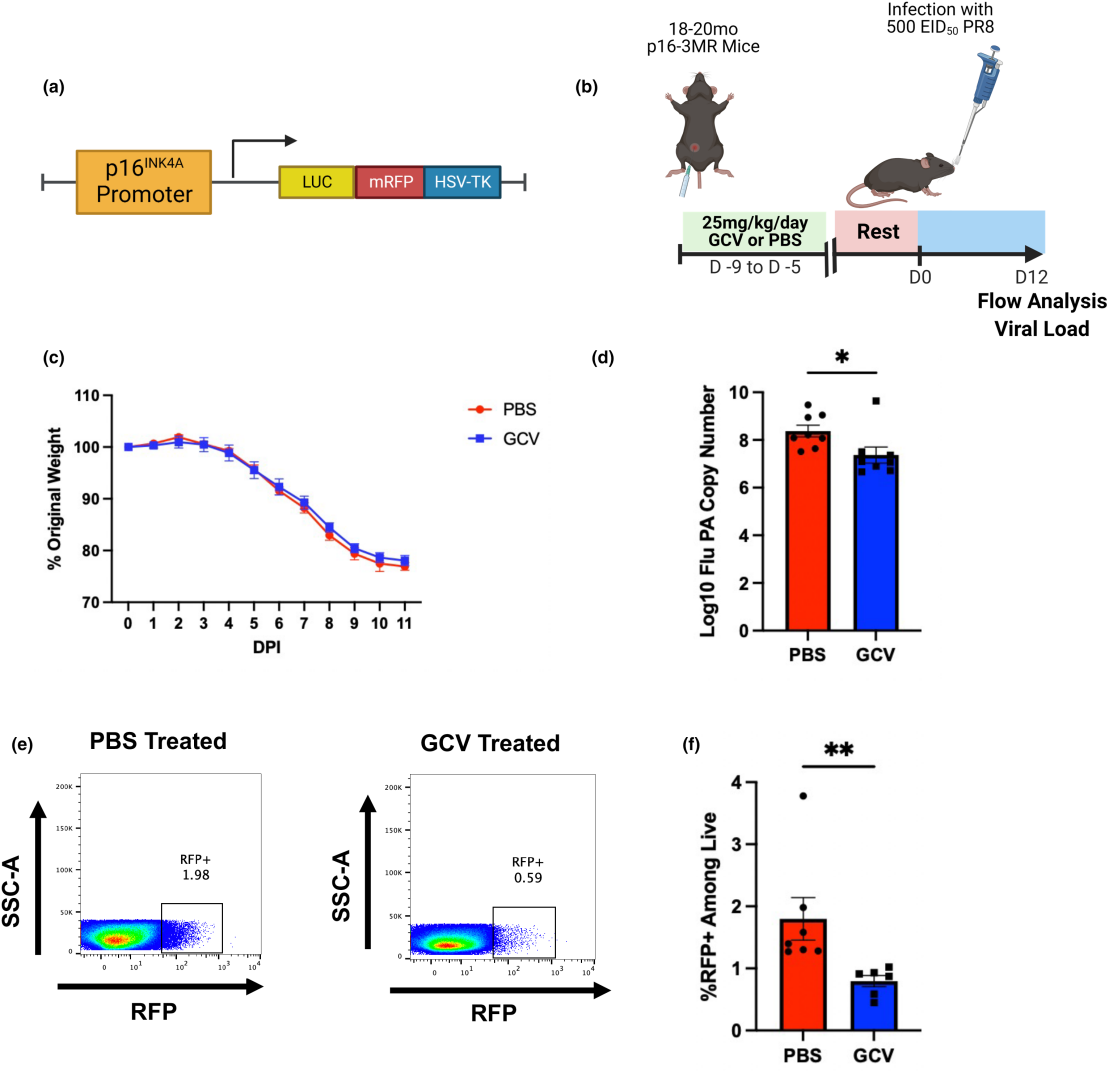
Targeting p16‐Expressing Cells is Effective in Potentiating the Primary Aged Antiviral Immune Response. The p16‐trimodality reporter (p16‐3MR) model expresses a fusion protein with three domains under the control of the p16^INK4A^ promoter: one expressing luciferase, another expressing mRFP, and another expressing the herpesvirus thymidine kinase (a). To study the effects of p16‐expressing cells on the aged immune response to influenza, we treated 18‐20mo p16‐3MR mice with 25 mg/kg/day ganciclovir (GCV) or PBS for 5 days. Following a 5 days rest period, we infected mice with sublethal dose of influenza (b). Percent of original weight lost was tracked throughout the course of infection with PR8 flu (c). At 12 days post infection (DPI) with PR8 flu, one cohort of mice was used to quantify viral load in the lungs via RT‐qPCR (d). RFP expression out of total live cells was quantified up to 30 DPI with x31 flu to confirm efficacy of GCV treatment (e,f). Data are presented as mean +/− standard error of the mean (SEM) and each symbol represents a single animal. Mann–Whitney *U*‐test was utilized for D and F, with a significance level of *p < 0.05; **p < 0.01. *N* = 6–8 per group (for experiments shown in B‐D, 3 males in GCV group and 4 males in PBS group. For experiments shown in E and F, 4 males in GCV group and 4 males in PBS group).

In this study, we utilized the p16‐3MR mouse model (Demaria et al., 2014) to examine the effects of p16‐expressing cells in both the primary and memory response to influenza. These mice express a fusion protein with three domains under the control of the p16 promoter: red fluorescent protein (mRFP), luciferase, and herpesvirus thymidine kinase (Figure 1a). These domains allow for identification of senescent cells via flow cytometry as well as selective clearance of these cells via administration of ganciclovir (GCV), a prodrug that reacts with the herpesvirus thymidine kinase to form an apoptosis‐inducing nucleoside analog causing DNA chain termination. While GCV is an antiviral drug, its mechanism of action is specific to herpesviruses that express thymidine kinase and thus no effects on flu infection were expected. Out of an abundance of caution, mice were rested for 5 days after GCV administration prior to flu infection. Other groups using this model have demonstrated effective clearance of p16‐expressing cells upon GCV administration (Patil et al., 2019). Studies utilizing this model have identified the presence of senescent cells in the lungs of aged p16‐3MR mice (Kaur et al., 2021), but this model has yet to be used to study the contribution of senescent cells to diminished immune responses after flu infection in aged mice. First, to investigate the effects of GCV treatment on senescent cells in the lungs of naïve mice, following GCV or PBS treatment, lungs were harvested from a group of aged (18–20 months old) p16‐3MR mice and cells were assessed for the senescence markers senescence‐associated beta‐galactosidase (SA‐β‐gal) and gamma‐H2AX (γ‐H2AX) (Figure S1). These 2 markers have been shown to be good indicators of senescence (Biran et al., 2017) and are now measurable by flow cytometry. In the CD45^+^ population (Figure S1a), we observed a trending reduction in the population expressing SA‐β‐gal (Q1) in the GCV‐treated group. We also saw a trending increase in the GCV‐treated population in the γ‐H2AX expressing population (Q3). These results indicate that it is likely that GCV treatment is targeting SA‐β‐gal expressing p16^+^ cells but not γ‐H2AX ^+^ p16^+^ cells within the CD45^+^ population. With regard to CD45^−^ lung cells, we found that the GCV‐treated group was trending lower in both Q1 and Q2, indicating that p16^+^ cells expressing SA‐β‐gal alone or in combination with γ‐H2AX were being targeted. While it is important to note that the results in none of the groups compared reached the traditional *p* value of <0.05, they do suggest that GCV treatment targeting clearance of p16^+^ cells in lungs of aged p163MR mice does have an impact on the presence of cells expressing these two senescence markers. To determine whether targeting these cells could potentiate the response to flu, we treated aged (18–20 months old) p16‐3MR mice intraperitoneally with either GCV or PBS daily for 5 days. Following a 5 day rest period after the last dose to ensure full elimination of GCV, mice were infected with a sublethal dose of H1N1 flu A/Puerto Rico/8/34 (PR8) (Figure 1b). While clearance of p16^+^ cells did not affect weight loss following infection, it did significantly enhance efficacy of viral clearance at 12 days post infection (DPI) (Figure 1c,d, respectively). Highlighting the efficacy of this model, abrogation of p16 expression persisted to our latest time point assessed, 30 days post infection with H3N2 flu A/HKx31 (x31) (Figure 1e,f).

**FIGURE 2:**
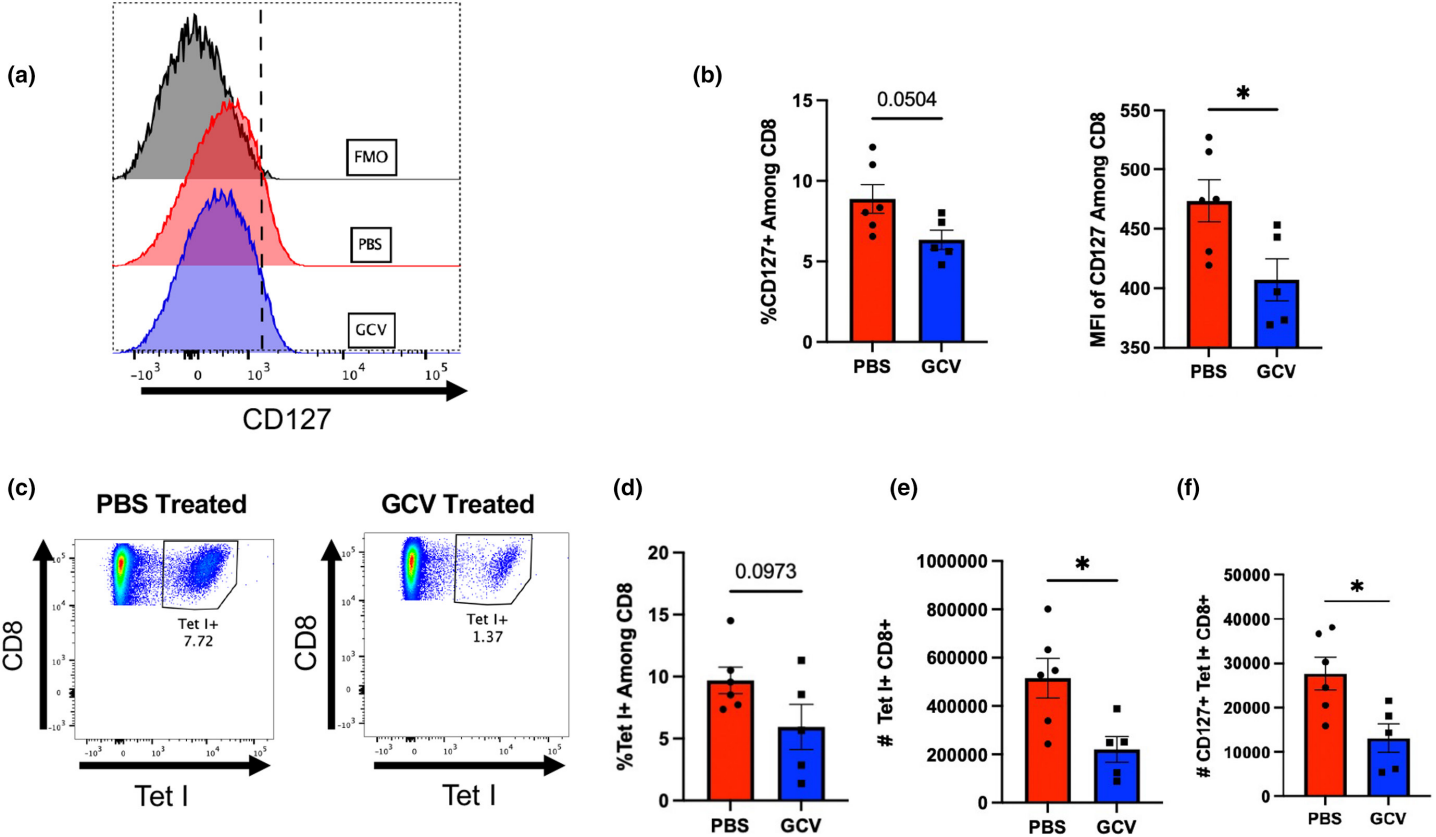
Targeting p16‐Expressing Cells Decreases Memory Precursor CD8 T cells in the Lung. At 12 DPI, lung‐infiltrating CD8 T cells were examined for surface CD127 (IL‐7Rα) expression. Both frequency of CD127+ CD8 T cells as well as mean fluorescence intensity (MFI) of CD127 among total CD8 T cells was quantified (a,b). Numbers of flu NP‐specific CD8 T cells (via influenza nucleoprotein MHC class I tetramer staining) and those expressing CD127 were also quantified (c‐f). All mice were infected using PR8. Data are presented as mean +/− standard error of the mean (SEM), and each symbol represents a single animal. Student's t‐tests were used for all comparisons with a significance level of *p < 0.05. *N* = 5–6 per group (3 males in GCV group and 4 males in PBS group).

To probe the mechanism of enhanced viral clearance, we first turned to the T‐cell compartment. Importantly, we sought to identify the balance of short‐lived effector T cells (SLECs) and memory precursor effector T cells (MPECs) via CD127 expression. CD127, or IL‐7 receptor alpha, is a key marker of MPECs (Kaech et al., 2003). IL‐7 is an important survival signal for memory T cells, MPECs expressing high levels of CD127 are more likely to survive following viral clearance and become memory cells equipped for a robust secondary response upon antigen re‐encounter. Lower CD127 expression is indicative of a population termed SLECs that are more effective at clearing an infection, but are more likely to die following resolution (Obar & Lefrançois, 2010). While typically a minority of effector T cells, MPECs have the greatest potential to differentiate into bona fide memory cells following resolution of infection. Clearance of p16‐expressing cells in aged p16‐3MR mice prior to flu infection resulted in a trending decrease in CD127‐expressing CD8 T cells in the lungs and significantly lower mean fluorescence intensity (MFI) of CD127 expression (Figure 2a,b, Complete gating strategy can be found in Figure S2). Interestingly, this phenomenon was limited to the site of infection and no changes in CD127 expression were seen in the spleen (Figure S3a,b). We also observed that GCV treatment induced a decrease in the number and of flu nucleoprotein (NP)‐specific CD8 T cells infiltrating into the lung at 12 DPI, while not affecting frequency (Figure 2c–e). While this may appear counterintuitive in light of the enhanced viral clearance in the GCV group, it most likely indicates that viral replication is already being well controlled and the decrease in numbers of flu‐specific cells is a means to control immunopathology. Further, enhanced viral clearance results in less antigen available to continue to stimulate immune responses. Similar to the total CD8 compartment, fewer flu NP‐specific CD8 T cells expressing CD127 were detected in the lungs of GCV‐treated mice (Figure 2f), although frequency of CD127^+^ cells among flu‐specific CD8 T cells was not changed (Figure S3c). Fewer NP‐specific CD8 T cells negative for CD127 were also observed in GCV‐treated mice (Figure S3d). However, the number of overall CD8 T cells was not changed (Figure S3e). Thus, our results suggest that clearance of p16‐expressing cells resulted in a bias toward short‐lived effector CD8 T cells in the lungs and away from memory precursors, perhaps via perturbations in CD127 expression. Very little expression of p16 (as measured by RFP expression via flow cytometry) was observed in CD4 T cells, CD8 T cells, or B cells in the PBS control groups (Figure S3h).

Interestingly, these effects were not found among total CD4s or flu‐specific CD4s (Figure S3f). In light of our previous studies examining the effects of senolytic drug treatment using a combination of dasatinib and quercetin (D + Q) on T‐cell differentiation following flu infection (Lorenzo et al., 2022), we also assessed the balance of flu‐specific FoxP3‐expressing regulatory T cells (Tregs) and GATA3‐expressing Th2 CD4 T cells and found no difference (Figure S3g). Importantly, this result with CD4 T cells highlights the differences observed when using various approaches to target senescent cells.

**FIGURE 3:**
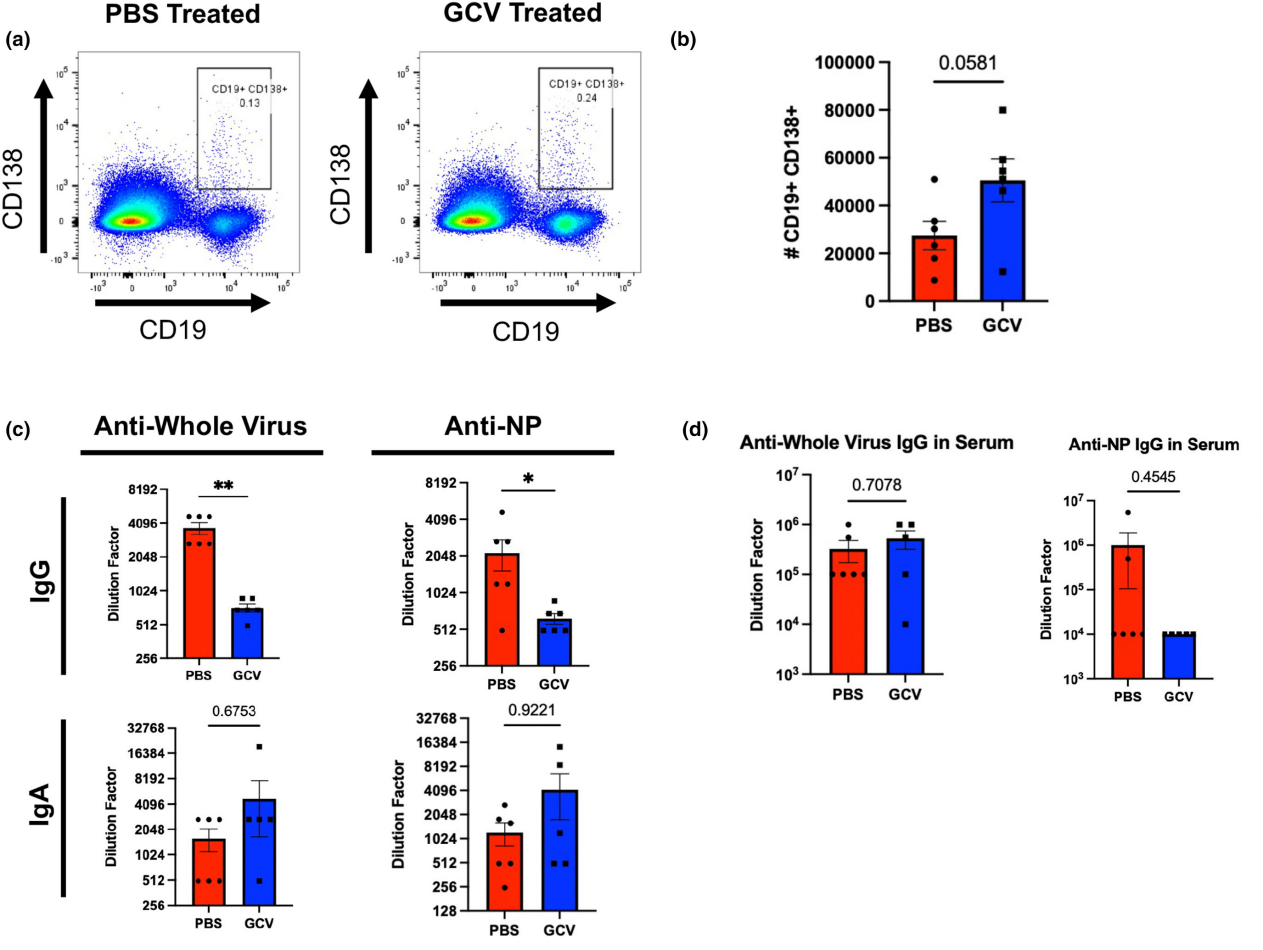
Targeting p16‐Expressing Cells Alters B‐cell Phenotypes and Antibody Secretion in the Lung. At 14 DPI, lung‐infiltrating B cells were identified as plasmablasts (CD19^+^ CD138^+^) and quantified (a,b). These were initially gated on CD4 and CD8 negative cells via a dump channel (as shown in Figure S4). Antibody titers were examined in bronchoalveolar lavage (BAL) to quantify IgA and IgG directed against either whole viral particles or flu nucleoprotein (NP) (c). IgG levels were quantified in the serum (d). All mice were infected using PR8. Data are presented as mean +/− standard error of the mean (SEM), and each symbol represents a single animal. Comparisons shown in B and top right panel of C were analyzed using Student's *t*‐test, all other comparisons were analyzed using the Mann–Whitney *U*‐test, all with a significance level of *p < 0.05; **p < 0.01. *N* = 5–6 per group (5 males in GCV group and 3 males in PBS group).

Examination of the B‐cell compartment revealed that p16 ablation did not induce any changes in class switched B cells in the lungs during flu infection (Figure S4b). However, we found that GCV treatment induced a nonsignificant increase in the number of CD19^+^ CD138^+^ plasmablasts (Figure 3a,b). This was limited to the lungs, and no similar increases in plasmablasts or CD19^−^ CD138^+^ plasma cells were observed in the spleen (Figure S4c). Despite trending increases in plasmablast numbers, virus‐specific IgG production in the bronchoalveolar lavage (BAL) was found to be sharply decreased in the GCV‐treated groups (Figure 3c). We assayed for IgG directed toward the whole viral particle itself as well as NP, an internal flu antigen that is highly conserved across strains and can confer broad protection (Lamere, Lam, et al., 2011; Lamere, Moquin, et al., 2011; Yewdell et al., 1985). By using the whole viral particle as a target, this approach includes quantification of a variety of neutralizing antibodies that could be directed toward any of the surface components of the particle including hemagglutinin (HA), neuraminidase (NA), and other external proteins. We also assayed for IgA directed toward these targets and did not observe any differences (Figure 3c). This phenomenon was limited to local antibody production, perhaps derived from responses occurring in the bronchus associated lymphatic tissue (BALT) since no significant deficits in IgG production were found systemically (Figure 3d). Thus, clearing p16‐expressing cells increased the overall number of antibody‐producing cells in the lung, but was associated with significant declines in the local concentration of antibody.

**FIGURE 4:**
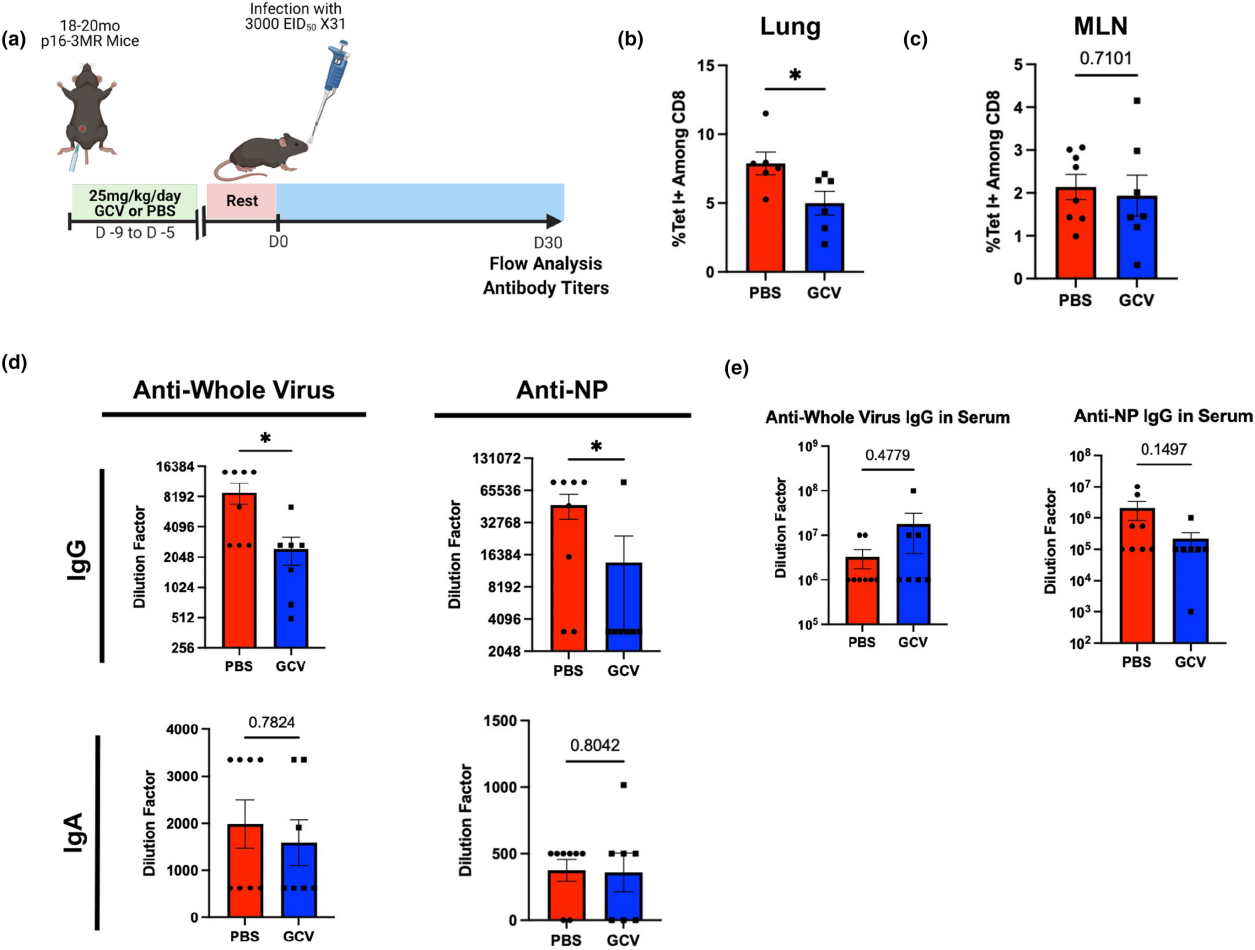
Targeting p16‐Expressing Cells Limits Development of Memory CD8 T cells and Antibody Levels in the Lung. Mice were treated as in (a). At 30 DPI, we assayed for flu NP‐specific CD8 T cells remaining in the lung (b) and the mediastinal lymph node (MLN) (c). Levels of IgG and IgA directed against whole viral particles as well as flu nucleoprotein (NP) were quantified in the bronchoalveolar lavage (BAL) (d). Serum IgG levels against both whole viral particles and NP were also quantified in the serum (e). All mice were infected using x31. Data are presented as mean +/− standard error of the mean (SEM). Comparisons shown in (b,c) were analyzed using Student's *t*‐test. (d,e) were analyzed using the Mann–Whitney *U*‐test, all with a significance level of *p < 0.05. An outlier above the mean in the PBS‐treated group in (b) was excluded for being more than 3 standard deviations from the mean. *N* = 6–8 per group (4 males in GCV group and 4 males in PBS group).

Effects of targeting p16‐expressing cells on immune memory formation and function the changes in CD127 expression that we observed during a primary infection led us to hypothesize that GCV treatment may alter memory CD8 T‐cell populations. To examine this, we treated aged p16‐3MR mice with either GCV or PBS. Following the rest period, mice were infected with a sublethal dose of x31 as a primary infection (Figure 4a). x31 flu was chosen as the primary infection for experiments focused on memory because it is less pathogenic than PR8 and allows for later rechallenge with PR8 to test the heterologous protection conferred by memory T cells. This schema has been commonly used to assess the protection conferred via heterosubtypic immunity (Powell et al., 2007). At 30 DPI, we assessed generation of memory CD8 T cells in the lungs, since these confer the most robust protection when flu is re‐encountered in the respiratory tract (Pizzolla et al., 2017). GCV‐treated mice showed a marked decrease in the frequency of flu‐specific memory CD8 T cells in the lungs corresponding with the observed reduction in CD127 expression (Figure 4b). No difference was observed in overall frequency of CD8 T cells nor in absolute numbers of flu‐specific CD8 T cells (Figure S5a,b, respectively). Interestingly, no difference was observed between the two groups in the mediastinal lymph node (MLN) (Figure 4c). Specific subsets of memory cells, T effector memory (Tem, CD44^+^ CD62L^−^), T central memory (Tcm, CD44^+^ CD62L^+^), and tissue resident memory (Trm, CD103^+^ CD69^+^) were not differentially affected by GCV treatment (Figure S5c). CD4 memory T cells were also unaffected (Figure S5d). Likely, this decrease in flu‐specific memory CD8 T cells is a consequence of the shift in favor of short‐lived effector functions at the cost of memory precursor differentiation.

Also at 30 DPI, similar to our results at 14 DPI (Figure 3), GCV treatment resulted in sharply decreased IgG levels in the BAL against whole virus and NP (Figure 4d). Because flu NP is so highly conserved, NP‐specific antibodies confer heterosubtypic cross‐protection much more robustly than antibodies directed toward external viral antigens such as HA or NA, which can be highly variable across strains. As before, effects of GCV treatment on antibody concentration were limited to the site of infection and systemic IgG levels were not significantly affected (Figure 4e).

**FIGURE 5:**
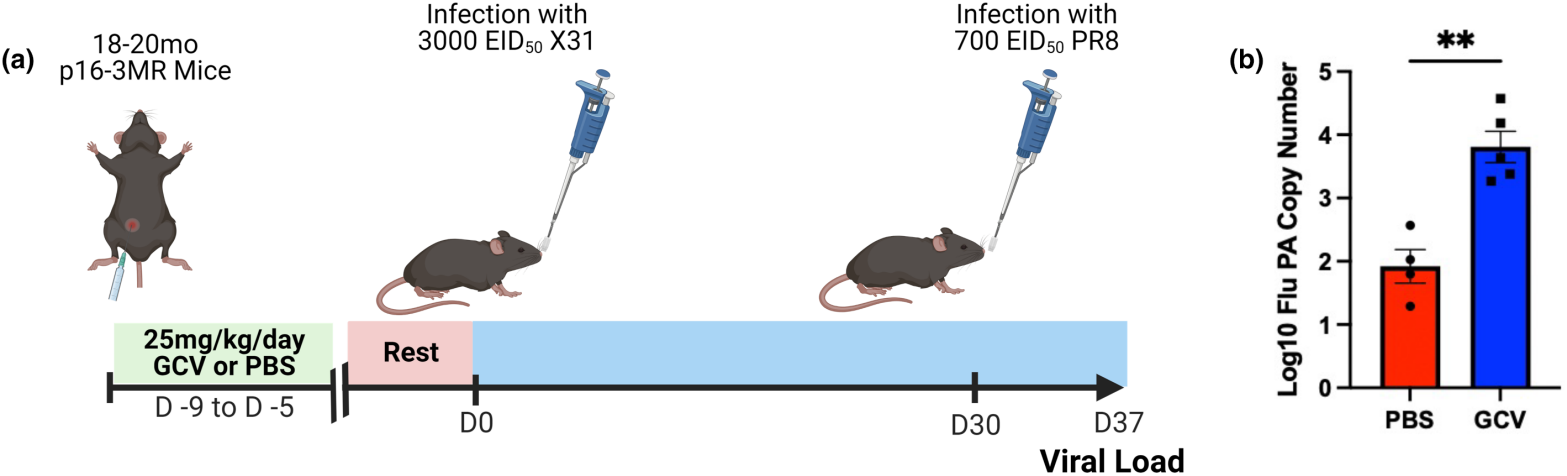
Clearance of p16‐Expressing Cells Prior to Infection Impairs the Protective Function of Immunological Memory Upon Pathogen Re‐encounter. Mice were treated with ganciclovir or PBS and subsequently infected with x31 flu at 30 DPI, mice were rechallenged with PR8 (a). At 7 days following rechallenge, viral load was quantified in the lungs via RT‐qPCR (b). Data are presented as mean +/− standard error of the mean (SEM), and each symbol represents a single animal. Comparisons were analyzed using Student's *t*‐test with a significance level of *p < 0.05; **p < 0.01. *N* = 4–5 per group (2 males in GCV group and 0 males in PBS group).

We hypothesized that while our observed deficit in memory T‐cell generation was moderate, the protection conferred upon pathogen re‐encounter would be deleteriously affected. To assess protection directly, we utilized the same GCV treatment schema as described previously (Figure 5a). In this case, at 30 DPI following x31 administration, we rechallenged with a sublethal dose of PR8 to test the protectiveness of the memory cells formed following the primary infection. Mice treated with GCV prior to the primary infection were less effective at clearing the virus compared to PBS‐treated controls (Figure 5b). Despite similar numbers in memory CD8 T cells in the lung (Figure S5b), the reduced protective capacity reveals a deficit in function of those memory cells formed in the absence of p16‐expressing cells. Therefore, our results suggest that p16‐expressing cells play a key role, perhaps through one or more SASP factors, in fostering the effector to memory transition in CD8 T cells.

## 4 DISCUSSION

This work reveals that the role of senescent cells in aging may be more complicated than previously appreciated. Cellular senescence may be related to the altered functionality of the aged immune system, as opposed to outright dysfunction. Immune responses are complex and require a delicate balance of pro‐ and anti‐inflammatory signals. When considering the physiological role of senescence, especially in the context of wound healing, it is possible that senescence may influence healing following an immune response. A key feature of healing is the effector to memory transition, the process by which memory precursors survive and differentiate into bona fide memory cells. Although the concept of immunological memory is foundational, the precise mechanisms by which memory cell fate is conferred are still largely unknown. Because these processes are multifaceted and involve various cell types and various soluble factors, approaches leveraging single cell‐resolution are likely the best course forward. Single cell technologies have begun to unravel these processes, but much remains to be determined (Buchholz et al., 2016). It is unclear in our model whether the functionality of these memory cells is affected or if it is simply a quantitative effect on their development. It is also unclear what mechanisms underly the changes we observe in the B‐cell compartment. In fact, the presence of tissue resident B cells in the lungs has only recently been observed (Allie et al., 2019; Lee & Oh, 2022). Very little is known whether and how the function of tissue resident memory B cells and antibody secreting cells changes with age, and future work will undoubtedly reveal additional complexities. Transcriptomic approaches may reveal important differences among both the T‐ and B‐cell compartments.

Our results here suggest that cellular senescence may play a role in the generation of immunological memory. It is important to note, however, that it remains unclear whether cellular senescence is involved directly. It is known that cytokine environments can alter the balance between memory precursor populations (Joshi et al., 2007), and this may be greatly influenced by SASP production in older animals. Because our results indicate deficits in both humoral and cellular memory, it is difficult to tease apart which of these are the most important in contributing to the loss of protection. Both of these mechanisms of immune memory are critically important to conferring protection, and it appears that senescent cells may be involved in the development both within the site of infection. All of these aspects of how senescence (including p16‐expressing cells) impacts adaptive immunity in the lung are continuing to be investigated in the Haynes laboratory and, we should have some novel insights in the next few years.

It is important to note that p16 expression alone is not a fully reliable identifier for senescent cells. While the p16‐expressing population certainly includes senescent cells, it may also include other cells that are not truly senescent. In our study, we do not think it likely that lymphocytes are targeted in any significant way due to lack of p16 expression among T and B cells in the lungs of our PBS control groups (Figure S3h). Quantification of SASP factor expression is a common way to ensure that senescent cells are being cleared; however, this proves difficult during an active immune response. Most SASP factors are proinflammatory cytokines that are commonly expressed during an infection in order to orchestrate the immune response. The study of senescent cells during immune responses thus poses particular challenges that have yet to be fully solved. However, as the field moves toward a canonical set of identifiers for senescent cells, these problems may be resolved.

Aside from SASP factors, it is also possible that clearing senescent cells may affect many populations, including structural cells such as epithelial or endothelial cells. With our study, it is not clear what cell types express p16 nor what tissues they reside in. Clearance of p16‐expressing cells in tissues other than the lung or lymph nodes could also alter responses in more systemic ways. Recently, p16‐expressing fibroblasts were found to be a critical population for maintaining barrier integrity and tissue remodeling in the lungs (Reyes et al., 2022). Senescent cells have also been shown to play a key role in limb regeneration in amphibian models (Walters et al., 2023). Clearance of these cells may compromise the barrier function of tissues and result in aberrant immune cell trafficking or leaking of certain cytokines or chemokines into the circulation. It is also possible that stromal cells responsible for organization of lymphoid tissues may be senescent and targeting them can exacerbate the age‐related changes in organization and delineation between T‐ and B‐cell zones required for optimal antibody responses. It is unclear whether the overall deficit in protection to heterosubtypic challenge is primarily mediated by the T‐cell changes or the changes in antibody secretion. It seems likely that senescent cells may be involved in the T‐ and B‐cell communication networks that promote robust antibody production. Further work may verify this, which would offer critical insight into the root causes of the declines in antibody production we observed in GCV treated aged p16‐3MR mice.

This work stands in contrast to our own recent report utilizing senolytics prior to a primary flu infection to clear senescent cells (Lorenzo et al., 2022). In that study, we found that D + Q administration to aged (18–20 months old) C57BL/B6 mice resulted in: (1) a significant reduction in TGF‐production in the lungs and Treg differentiation, and (2) no impact on the generation of effector CD8 T‐cell subsets in the lungs during the primary response. Interestingly, a study utilizing fisetin, another senolytic drug, found that targeting senescent cells improved serum antibody production (Camell et al., 2021). This highlights the heterogeneity and diversity of senescent cells and the cell types preferentially targeted by different senolytic drugs. Two recent studies have identified the ability of tissue resident macrophages in murine lungs to take on a senescent phenotype (Haston et al., 2023; Prieto et al., 2023). Tissue resident macrophages and other professional antigen presenting cells are important for orchestrating CD4 T‐cell responses. Perhaps D + Q targets macrophages more readily than the p16‐3MR approach. A recent report has illustrated senescent cell type diversity by concluding that certain aging phenotypes are driven primarily by senescent cells expressing p21^Cip1^ but not p16 (Chandra et al., 2022). Differences between models could help further explain discrepancies where most senolytic drugs are delivered via oral gavage and have differing pharmacokinetics than our approach delivering GCV intraperitoneally. This may result in varying degrees of senescent cell clearance and may be a confounding variable when comparing different studies. There are also known sex differences in immune responses to influenza (Jacobsen & Klein, 2021), which need to be explored further in our models.

It is also important to fully characterize both primary and secondary responses when assessing the function of the adaptive immune system's ability to combat a pathogen. The regulation of the kinetics of an immune response is dynamic and perturbations in cell populations must be probed across time, which has not yet been done in the context of targeting senescent cells to alter aged immune responses. Many outstanding questions remain that could not be completely addressed by this study: (1) what is the role of senescent cells in a young immune response? (2) what SASP factor is responsible for altering T‐cell memory precursor differentiation? (3) how is the senescence environment altering the function of antibody production by B cells? Our results bring to light potential concerns with the use of senolytics to potentiate immune responses in older adults. If p16‐expressing cells play a role in shaping the formation of durable and protective memory with age, it may be detrimental to target senescent cells with the aim to improve vaccine responses. It also is unclear how persistent the effects of clearing senescent cells are and how long it would take for senescent cells to reappear if typical aged immune responses would return as well. Clinical trials are ongoing to study effects of senolytics on COVID‐19 disease severity in older adults, and it will be helpful to understand how this approach may affect differentiation of memory T cells and antibody production following recovery (NCT04476953). This, combined with further mechanistic studies in animal models, may help us to better understand cellular senescence and its effects on susceptibility to infection, generation of immunological memory, general immune function, and other processes of aging.

## AUTHOR CONTRIBUTIONS

BLT planned and conducted experiments, led data analysis, and wrote the first draft of the manuscript. ANC, HAP, DEM, and ECL assisted with conducting experiments. ERJ, ECL, and JMB assisted with designing experiments and provided guidance on data analysis. LH was the principal investigator for this study and acquired funding as well as provided mentorship and guidance for planning experiments and data interpretation. All authors read and edited the manuscript.

## CONFLICT OF INTEREST STATEMENT

The authors declare this research was completed in the absence of any potential conflicts of interest.

## Supporting information


Appendix S1.

Data S1.

## Data Availability

All data supporting this manuscript will be made available by the corresponding author upon reasonable request.
