# Supplementary material for: Cellular senescence is a double‐edged sword in regulating aged immune responses to influenza
Source: Aging Cell. 2024 Apr 30;23(7):e14162. doi: 10.1111/acel.14162 (PMC11258475; doi:10.1111/acel.14162)

Supplemental Table 1. Extended Antibody Information for Flow Cytometry Experiments

| Specificity | Fluorochrome | Clone | Dilution | Vendor/Supplier |
| --- | --- | --- | --- | --- |
| CD4 | BV650 | RM4-5 | 1:100 | BD Biosciences |
| CD4 | BUV737 | RM4-5 | 1:300 | BD Biosciences |
| CD8a | BUV737 | 53-6.7 | 1:100 or 1:300 | BD Biosciences |
| NP311-325 IAb MHC Class II tetramer | BV421 | N/A | 1:50 | NIH Tetramer Core |
| NP366-374 H-2Db MHC Class I tetramer | APC | N/A | 1:50 | NIH Tetramer Core |
| CD127 | APC-Cy7 | AZR34 | 1:100 | Biolegend |
| Tbet (Intracellular) | BV711 | 4B10 | 1:100 | Biolegend |
| FoxP3 (Intracellular) | AF700 | FJK-16s | 1:100 | ThermoFisher |
| GATA3 (Intracellular) | FITC | 16E10A23 | 1:200 | Biolegend |
| Bcl6 (Intracellular) | PerCPef710 | BCL-DWN | 1:1000 | ThermoFisher |
| CD44 | FITC | IM7 | 1:100 | BD Biosciences |
| CD62L | APC-Cy7 | MEL14 | 1:100 | BD Biosciences |
| CD127 | BV711 | AZR34 | 1:100 | Biolegend |
| CD69 | BV605 | H1.2F3 | 1:100 | BD Biosciences |
| CD103 | PerCP-Cy5.5 | 2E7 | 1:100 | Biolegend |
| CD19 | BV650 | 6D5 | 1:200 | Biolegend |
| CD138 | PE-Cy7 | 281-2 | 1:100 | Biolegend |
| IgD | APC | 11-26c | 1:300 | ThermoFisher |
| IgM | FITC | 11/41 | 1:300 | BD Biosciences |
| yH2aX | AF647 | 2F3 | 1:100 | BioLegend |
| β-galactosidase | Absorption/emission: 490/514 nm | CellEvent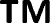 Senescence Green  Detection Kit | | Invitrogen |

# A)

| Q1 | Q2 |
| --- | --- |
| Q4 | Q3 |

CD45+ Q1 CD45+ Q2

PBS

GCV

0

2

4

6

8

10

% of CD45

-

population

0.1268

PBS

GCV

0

20

40

60

80

100

% of CD45

-

population

0.2074

SA-

b

gal

-

| B) | CD45- Q1 | CD45- Q2 |
| --- | --- | --- |

## g-H2AX

PBS

GCV

0.00

0.05

0.10

0.15

0.20

% of CD45

-

population

0.1820

PBS GCV

0.0

0.5

1.0

1.5

2.0

2.5

% of CD45

-

population

0.0909

PBS

GCV

0

1

2

3

% of CD45

+

population

0.1114

PBS

GCV

0.00

0.05

0.10

0.15

0.20

0.25

% of CD45

+

population

0.3129

| CD45+ Q4 | CD45+ Q3 |
| --- | --- |

PBS

GCV

0

5

10

15

20

25

% of CD45

+

population

0.0954

GCV

PBS

0

20

40

60

80

100

% of CD45

+

population

0.1935

| CD45- Q4 | CD45- Q3 |
| --- | --- |


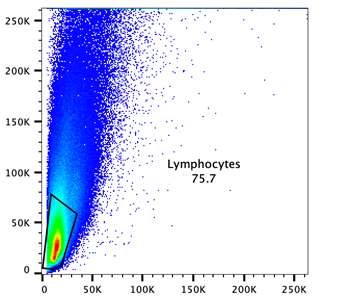

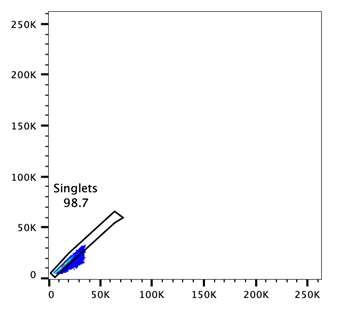

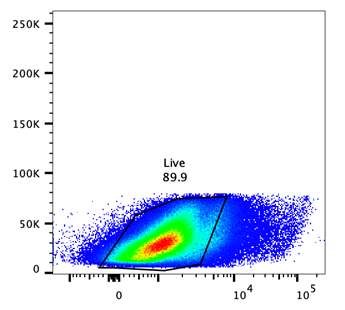

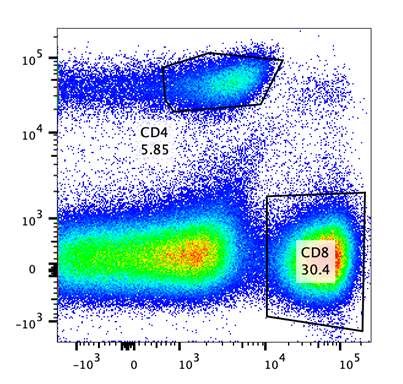

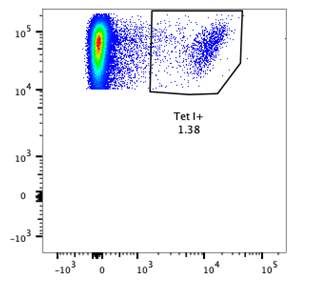

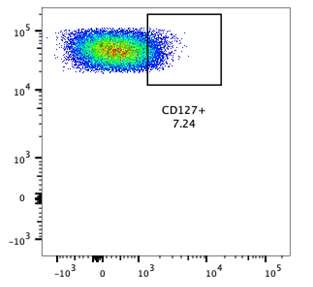

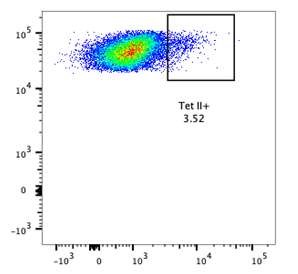


FSC-A

SSC-A

FSC-A

FSC-H

SSC-A

Live/Dead-UV

CD4-BV650

CD8-BUV737

CD4-BV650

CD4-BV650

CD127-APC-Cy7

CD8-BUV737

Tet I-APC

Tet II-BV421


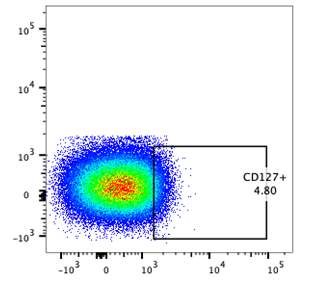


CD4-BV650

CD127-APC-Cy7

A) B)


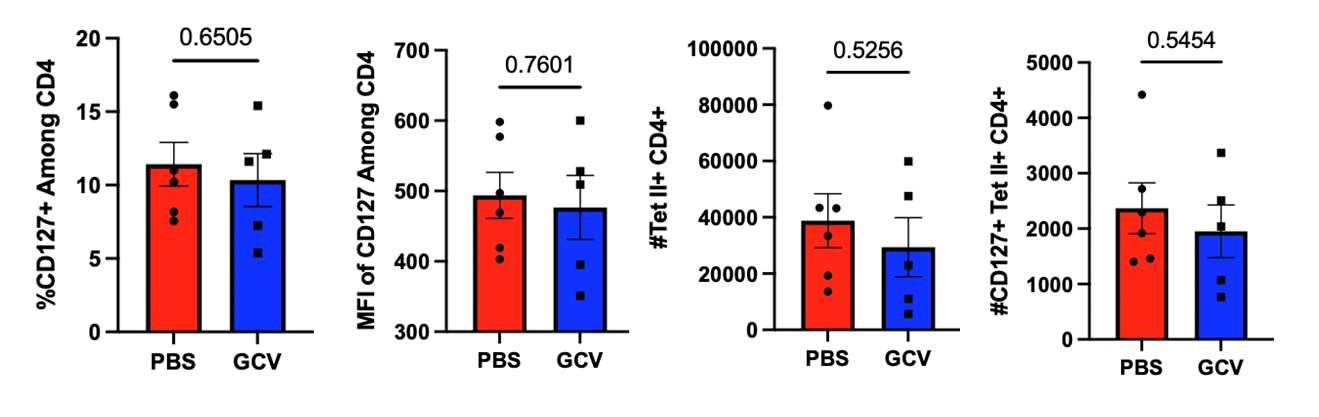


CD127

F)


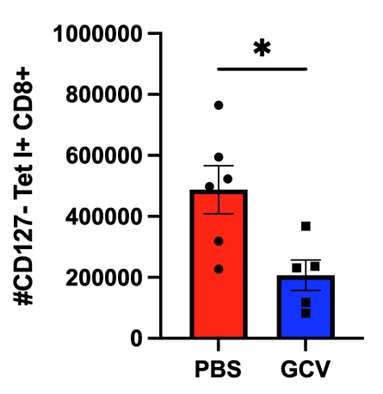


E)


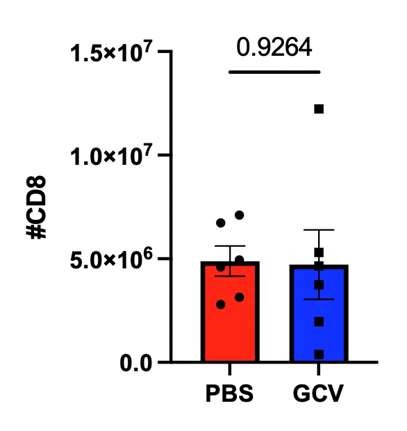


C)

PBS GCV

0

2

4

6

8

10


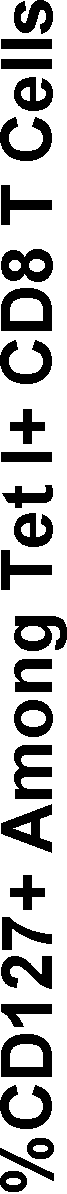


0.5962

D)


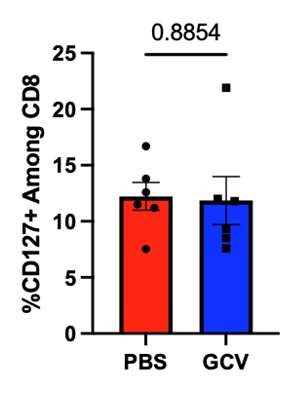

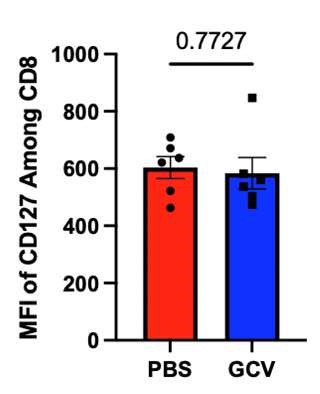

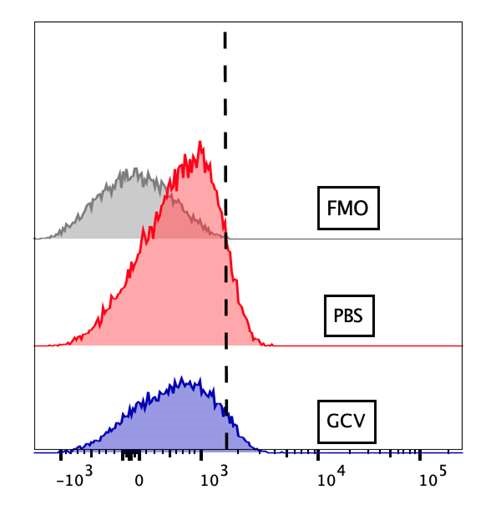


# G)
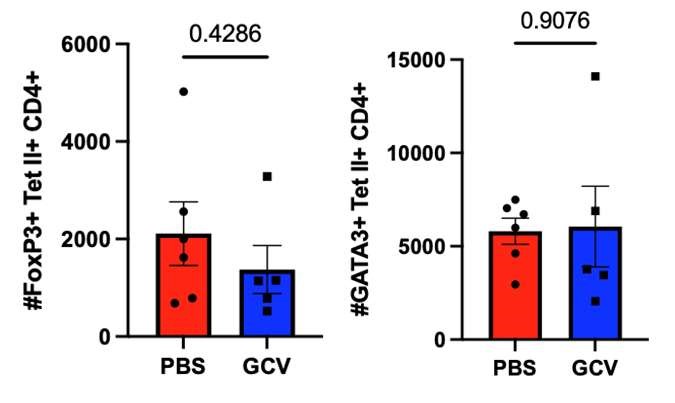
 H)
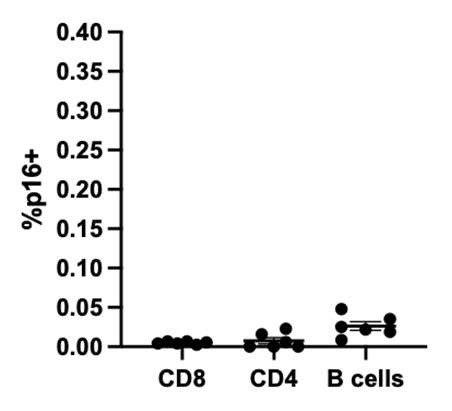


1. Dump: CD4, CD8


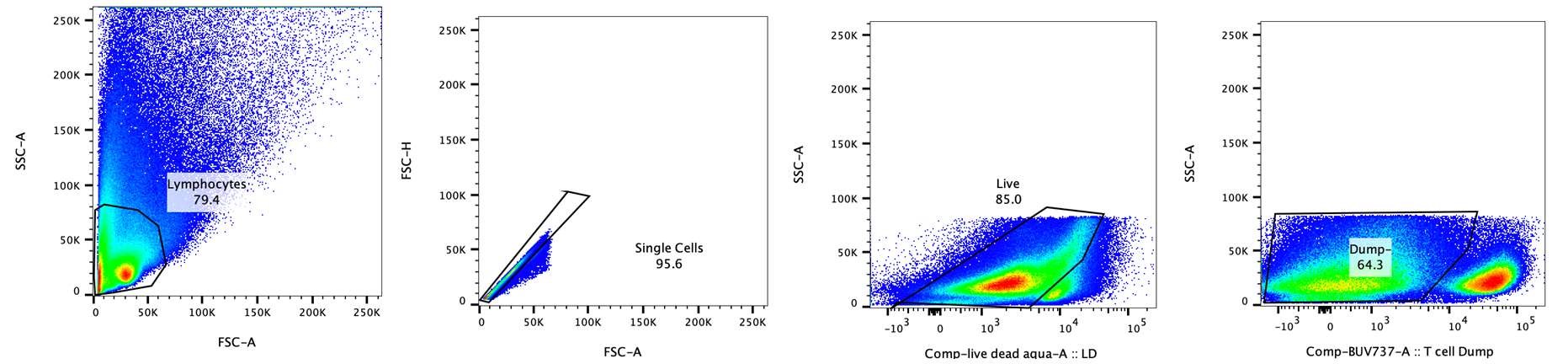

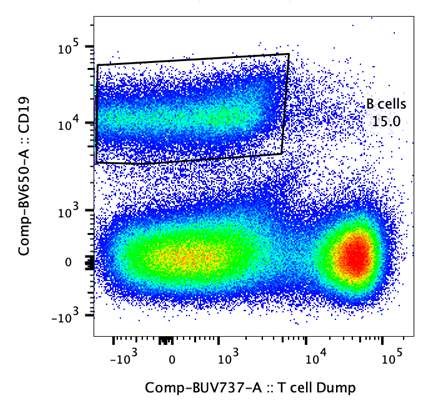

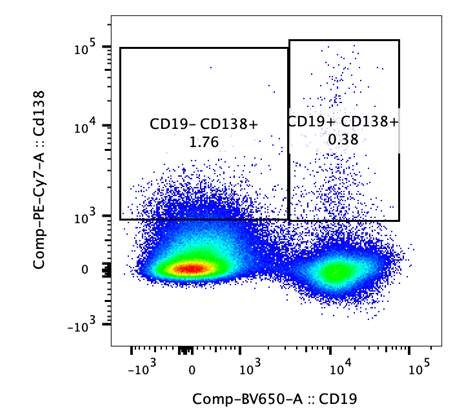

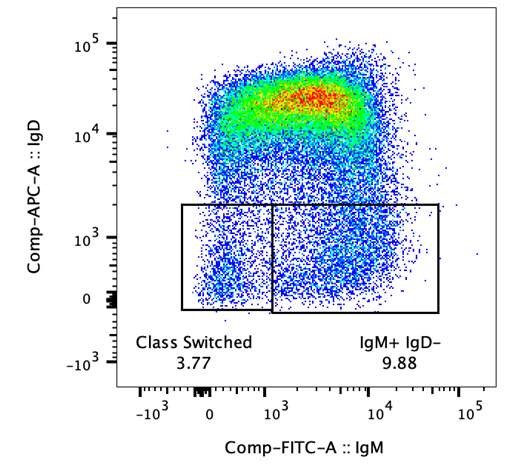


FSC-A

SSC-A

FSC-H

FSC-A

SSC-A

Live/Dead-Aqua

Dump-BUV737

SSC-A

CD19-BV650

CD138-PE-Cy7

Dump-BUV737

CD19-BV650

IgM-FITC

IgD-APC

1. C)


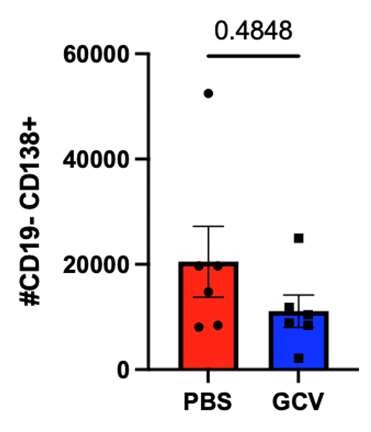

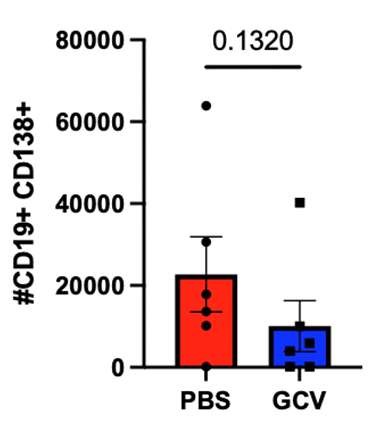

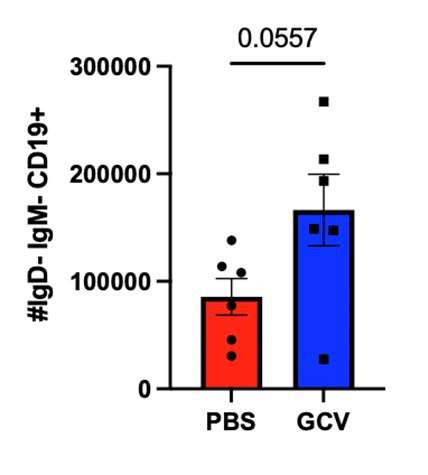

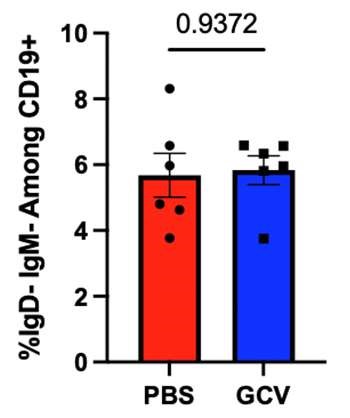


# A)B)


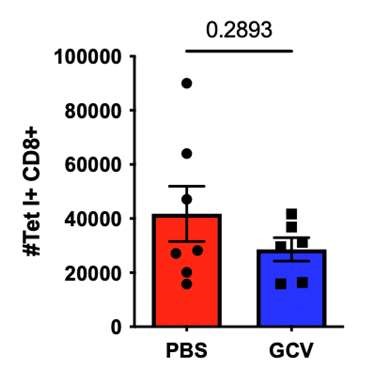

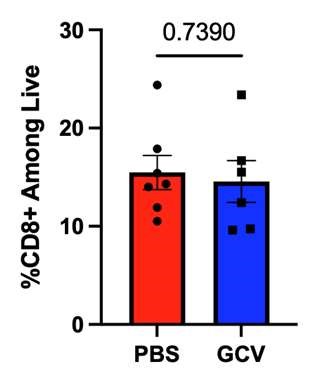


# D)


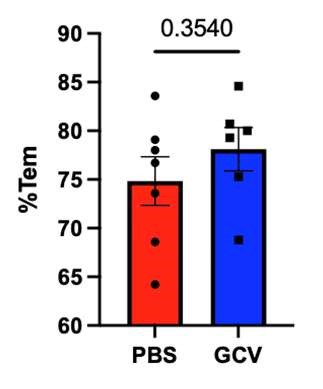

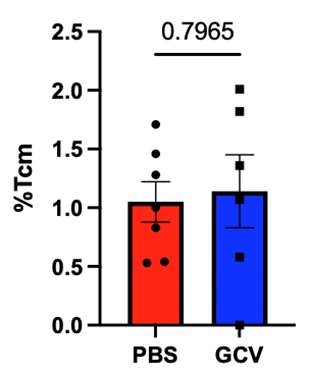

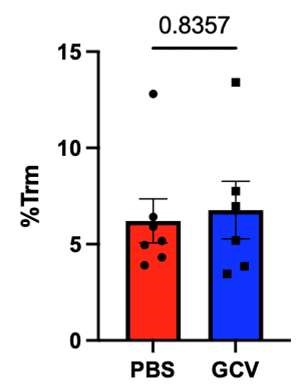

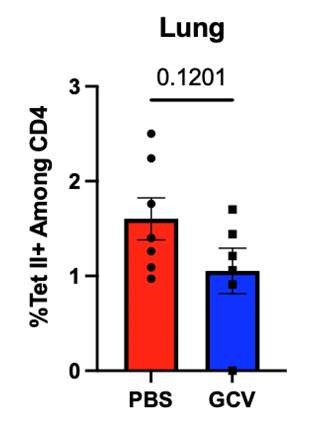

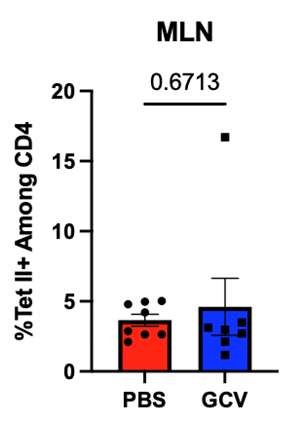

Supplement: Supplementary file 1 — Appendix S1. [file ACEL-23-e14162-s001.docx]
