# Supplementary material for: Cellular senescence is a double‐edged sword in regulating aged immune responses to influenza
Source: Aging Cell. 2024 Apr 30;23(7):e14162. doi: 10.1111/acel.14162 (PMC11258475; doi:10.1111/acel.14162)
Supplement: Supplementary file 2 — Data S1. [file ACEL-23-e14162-s002.docx]

Supplemental Figure S1. Expression of Senescent Cell Markers on Lung Cells After GCV Treatment.
Naïve (uninfected) 18 mo. Old p16-3MR mice (N = 3/group) were treated with GCV or PBS for 5 days as described in methods. Five days after the last treatment, lungs were harvested and digested for flow cytometry phenotyping for expression of the senescence markers senescence-associated beta-galactosidase (SA-β-gal) and gamma-H2AX (γ-H2AX). Panel A shows the percent of CD45+ lung cells and panel B shows CD45- lung cells in each of the 4 flow dot plot quadrants (delineated in mock flow cytometry dot plot at right). GCV treated and PBS control groups were compared by Student's t-test and p values for each graph are shown.
Supplemental Figure S2. Flow Cytometric Analysis of Primary T cell Responses All T cell phenotyping at primary time points utilized the gating strategy shown.
Supplemental Figure S3. Alterations in Primary T cell Responses in the Absence of p16- Expressing Cells are CD8 Specific and Limited to the Lungs
At 12 DPI, CD8 T cells in the spleen were assayed by flow cytometry for expression of CD127 as in Figure 1 (A and B). Frequency of CD127+ cells among Tet I+ CD8 T cells was measured (C). Absolute number of CD127- Flu-specific CD8 T cells were measured (D). Total number of CD8 T cells in the lungs were quantified by flow cytometry. (E). At 12 DPI, lung-infiltrating CD4 T cells were identified as memory precursors in the general CD4 and Flu NP-specific compartment (F). FoxP3 and GATA3 expression were quantified among Flu NP-Specific CD4s in the lung (G). In the lungs, p16 expression was assessed via RFP expression in PBS-treated aged p16-3MR mice within CD4 T cells, CD8 T cells, and B cells in at 12 DPI (H). Data are presented as mean +/− standard error of the mean (SEM) and each symbol represents a single animal. Left panel of G was analyzed using Mann–Whitney U-test. All other comparisons were analyzed using Student's t-test with a significance level of p < 0.05. N = 5–6 per group (3 males in GCV group and 4 males in PBS group).
Supplemental Figure S4. Flow Cytometric Analysis of B cell Phenotypes Following Flu Infection
All B cell phenotyping utilized the gating strategy shown in A. A dump channel containing anti-CD4 and CD8 was used. At 14 DPI, B cells in the lungs were assayed for expression of IgD and IgM to determine class switched status (B). At 14 dpi, spleen plasmablasts (CD19+ CD138+) and plasma cells (CD19− CD138+) were quantified (C). Data are presented as mean +/− standard error of the mean (SEM) and each symbol represents a single animal. Comparisons in the left panel of B and all of C were analyzed using Mann–Whitney U-test. All other comparisons were analyzed using Student's t-test with a significance level of p < 0.05. N = 6 per group (5 males in GCV group and 3 males in PBS group).
Supplemental Figure S5. Alterations in Generation of T cell Memory in the Absence of p16-Expressing Cells are CD8 Specific and Do Not Affect Memory Subsets
Mice were infected with sublethal dose of X31 H3N2 flu. At 30 DPI, frequency of CD8 T cells overall (A) and number of Flu NP-specific CD8 T cells in the lung were quantified (B) and classified into memory subsets of T effector memory (Tem, CD44+ CD62L-), T central memory (Tcm, CD44+, CD62L+) and tissue resident memory (Trm, CD103+ CD69+) (C). Frequency of Flu NP-specific CD4 T cells was also assessed in the lung and mediastinal lymph node (MLN) (D). Data are presented as mean +/− standard error of the mean (SEM) and each symbol represents a single animal. Comparison in the middle panel of B and right panel of C were analyzed using Mann–Whitney U-test. All other comparisons were analyzed using Student's t-test with a significance level of p < 0.05. N = 6–7 per group (4 males in GCV group and 4 males in PBS group).
